# Medication for Opioid Use Disorder and Treatment Retention Among Pregnant Individuals

**DOI:** 10.1001/jamanetworkopen.2025.6069

**Published:** 2025-04-21

**Authors:** Valerie S. Ganetsky, Noa Krawczyk, Alene Kennedy-Hendricks

**Affiliations:** 1 Department of Health Policy and Management, Johns Hopkins Bloomberg School of Public Health, Baltimore, Maryland; 2 Center for Opioid Epidemiology and Policy, Department of Population Health, NYU Grossman School of Medicine, New York, New York

## Abstract

**Importance:**

Treatment retention for pregnant individuals with opioid use disorder (OUD) is critical, especially during the high-potency synthetic opioid (HPSO) era. Current data on the relationship between medication for opioid use disorder (MOUD) receipt in specialty substance use treatment facilities and retention are needed for this population.

**Objective:**

To examine the association between MOUD inclusion in treatment and 6-month treatment retention among pregnant individuals with OUD in publicly funded specialty treatment facilities during the HPSO era.

**Design, Setting, and Participants:**

This cross-sectional study pooled data from January 1, 2015, to December 31, 2021, from the Treatment Episode Data Set-Discharges, a national dataset managed by the Substance Abuse and Mental Health Services Administration that tracks annual discharges from state-licensed, publicly funded substance use treatment facilities. Individuals who were pregnant at the time of admission, reported an opioid (heroin, nonprescription methadone, or other opiates and synthetics) as their primary substance, and were discharged from ambulatory, nonintensive outpatient facilities were included. Data were analyzed November 2023 to April 2024.

**Exposure:**

MOUD inclusion in a treatment episode.

**Main Outcomes and Measures:**

The main outcome was treatment retention (length of stay >6 months vs ≤6 months). To account for the nonrandom assignment to MOUD, inverse probability of treatment–weighted logistic regression models were estimated adjusting for sociodemographics; substance use, mental health, and treatment history; treatment admission–related variables; census division; state policy characteristics; and year fixed effects.

**Results:**

Of 29 981 treatment episodes, most involved individuals aged 25 to 34 years (19 106 [63.7%]). Approximately two-thirds of 29 071 episodes in the final analysis (19 884 [68.4%]) included MOUD across all study years. From 2015 to 2021, MOUD inclusion in treatment episodes increased by 9.1 percentage points, from 65.0% to 74.1%. Treatment episodes with MOUD were associated with greater odds of 6-month treatment retention compared with those without MOUD (adjusted odds ratio, 1.86 [95% CI, 1.72-2.01]). This finding translated to an estimated 14.2 percentage point greater adjusted probability of 6-month retention among treatment episodes with MOUD (43.1%) vs those without it (28.9%).

**Conclusions and Relevance:**

In this cross-sectional study of treatment episodes from ambulatory, nonintensive facilities, MOUD inclusion among pregnant individuals was associated with significant improvements in treatment retention. However, retention remained low during the HPSO era. These findings underscore the importance of MOUD in improving OUD-related outcomes in this high-risk population.

## Key Points

**Question:**

Is there an association between medication for opioid use disorder (MOUD) receipt and treatment retention among pregnant individuals receiving care in publicly funded specialty treatment facilities during the high-potency synthetic opioid era?

**Findings:**

In this cross-sectional study of 29 981 treatment episodes, MOUD inclusion in treatment increased from 65.0% in 2015 to 74.1% in 2021. Adjusted probability of 6-month treatment retention was greater for MOUD episodes (43.1%) than for episodes without MOUD (28.9%).

**Meaning:**

The findings suggest that MOUD inclusion in treatment episodes among pregnant individuals may be associated with greater treatment retention during the high-potency synthetic opioid era.

## Teaser

This cross-sectional study examines the association between medication for opioid use disorder receipt and treatment retention for opioid use disorder among pregnant individuals during the high-potency synthetic opioid era.

## Introduction

Opioid use disorder (OUD) during pregnancy is a critical public health issue. Overdose, a leading cause of pregnancy-related death, increased by 81% between 2017 and 2020 among pregnant and postpartum populations, with rates nearly tripling among those aged 35 to 44 years between 2018 and 2021.^1,2,3^ Untreated OUD during pregnancy is associated with lower engagement with prenatal care and increased risk of preterm birth, low infant birth weight, and neonatal opioid withdrawal syndrome.^4^

Treatment involving medications for opioid use disorder (MOUD), including buprenorphine and methadone, is the gold standard of care for OUD during pregnancy.^4,5^ Use of MOUD is associated with reduced opioid-related overdose rates during the pregnancy and postpartum periods, improved adherence to prenatal care, and decreased risk of preterm birth and low infant birth weight.^6,7,8^ Given the maternal and neonatal benefits of MOUD, retaining pregnant individuals in treatment is critical. Longer durations of MOUD use during pregnancy are also associated with greater postpartum treatment engagement.^9^ Treatment retention has grown even more urgent during the era of fentanyl and other high-potency synthetic opioids (HPSOs) in the illicit drug supply. Notably, there has been a large increase in pregnancy-associated overdose deaths involving HPSOs.^3^ Given the role of HPSOs in influencing current overdose patterns among pregnant individuals, initiation of MOUD and retention in treatment are critical public health goals.

The OUD cascade-of-care framework recognizes that MOUD initiation and long-term treatment retention are crucial steps on the pathway between OUD diagnosis and recovery.^10^ Applying this framework to pregnant individuals with OUD is important to inform future clinical and policy efforts that seek to improve outcomes for this vulnerable population. Despite the clear benefits of MOUD, only an estimated 50% to 60% of pregnant individuals with OUD initiate MOUD.^11,12^ Prior research examining the association of MOUD with treatment retention among pregnant individuals is mostly limited to claims-based studies and periods preceding the proliferation of fentanyl across the illicit drug supply in the US, which has been associated with dramatically increased overdose rates.^6,9,13,14,15^ More research is needed to assess the relationship between MOUD receipt and treatment retention among pregnant populations in more recent years using data sources with national reach. Furthermore, the prior literature mainly focused on factors associated with MOUD receipt (vs the impact of MOUD on treatment retention) and did not use methods to address nonrandom selection of pregnant individuals into MOUD treatment when assessing the association between MOUD receipt and outcomes, such as treatment retention.^12,16,17,18^ This analysis used the most current national data on specialty substance use treatment program discharges among pregnant individuals and used robust methods to address baseline differences among those who did and did not receive MOUD to examine the association between MOUD and treatment retention.

## Methods

### Study Design and Data Source

This cross-sectional study used pooled data from January 1, 2015, to December 31, 2021, from the Treatment Episode Data Set-Discharges (TEDS-D), a national dataset managed by the Substance Abuse and Mental Health Services Administration that tracks annual discharges from state-licensed substance use treatment facilities that receive federal public funding. TEDS-D provides treatment episode–level data from facilities’ administrative records that are reported to the state. This study was deemed not human participants research by the Johns Hopkins Bloomberg School of Public Health institutional review board and did not require informed consent, as it used publicly available, deidentified, secondary data. The study was reported following the Strengthening the Reporting of Observational Studies in Epidemiology (STROBE) reporting guideline for cross-sectional studies.

### Study Sample

We included all treatment episodes for individuals who were pregnant at the time of admission, reported opioids (heroin, nonprescription methadone, or other opiates and synthetics) as their primary substance, and were admitted to ambulatory, nonintensive outpatient facilities. We included only ambulatory, nonintensive facilities as these are the settings where longer-term MOUD treatment is most likely to occur. We excluded treatment episodes with missing information on MOUD inclusion in treatment.

### Main Outcomes and Measures

Outcomes were measured at the treatment episode level. The primary outcome was treatment retention longer than 6 months based on the length of treatment episode variable, calculated for each episode using the admission date and the last contact date. The 6-month retention period was chosen because this time frame is recognized as an important quality metric of treatment continuity by the National Quality Forum.^19^ Inclusion of MOUD (methadone, buprenorphine, and/or naltrexone) in the treatment episode was the independent variable of interest.

Covariates hypothesized as potential confounders of the association between MOUD inclusion in the treatment episode and retention included the following: sociodemographic characteristics, including age, race and ethnicity, marital status, educational level, employment status, and housing; substance use, mental health, and treatment history, including route of opioid administration, secondary substance use, co-occurring mental health conditions, and any prior treatment episode; and treatment admission-related variables, including treatment referral source and treatment services provided at admission excluding detoxification. TEDS-D reports on race using the 5 minimum categories recommended by the Office of Management and Budget: American Indian or Alaska Native; Asian; Black or African American; Native Hawaiian or Other Pacific Islander, and White. Other single race included individuals not included in those categories. TEDS-D also includes records from states that do not separate the race categories of Asian and Native Hawaiian or Other Pacific Islander. We reported ethnicity as Hispanic and non-Hispanic. A detailed description of the values for each of the variables is available in eAppendix 1 in Supplement 1. We did not have sufficient sample size in all states to include state fixed effects; therefore, we included a 10-category census division variable to account for variation in treatment system infrastructure across the US. Given that Medicaid expansion is associated with increased MOUD receipt,^20,21^ we included a state-year indicator variable capturing state Medicaid expansion status in each year. Given that punitive policies targeting pregnant individuals with OUD may discourage treatment engagement,^22^ we also included a state-year indicator variable capturing whether the state had a law classifying prenatal substance exposure as child abuse or neglect in the year of the treatment episode.^23,24^ Finally, we included year fixed effects to account for variation in treatment practices across study years. Patient insurance type was not included in the analyses given a high level of missingness (61.9%).

### Statistical Analysis

First, we calculated the percentage of treatment episodes involving MOUD in each year of the study (2015-2021). Next, to estimate the association between MOUD inclusion in the treatment plan and treatment retention, we estimated inverse probability of treatment (IPT)–weighted logistic regression models.^25^ These IPT weights allowed us to weight more heavily in the main regression model for treatment episodes that did not include MOUD but were similar on observed characteristics to episodes that did include MOUD (eAppendix 2 in Supplement 1). To assess whether the IPT weighting succeeded in generating more comparable groups on observable characteristics, we calculated unweighted and IPT-weighted standardized mean differences for each covariate. An absolute difference of less than 0.2 for the weighted standardized mean difference indicated improved covariate balance after weighting.^26^ We then used these IPT weights to estimate a logistic regression model including all the aforementioned covariates for doubly robust estimation to examine the association between MOUD inclusion and treatment retention longer than 6 months. To quantify how strong an unobserved confounder would need to be to fully explain away the observed treatment effect, we calculated an E-value.^27^ We also explored potential modifications of associations by all covariates using interaction terms.

Following estimation of the weighted logistic regression models, to improve interpretability, we used postestimation margins, holding covariates at their means, to calculate adjusted estimated probabilities of treatment retention longer than 6 months among treatment episodes with or without MOUD and the marginal effect estimate (ie, the difference between these estimated probabilities) for MOUD and treatment retention.

We performed several sensitivity analyses. First, we estimated models excluding treatment episodes with 1 or more prior episodes to account for multiple admissions during our study period. Second, we excluded treatment episodes during 2020 and 2021 to account for possible changes in treatment use patterns during the COVID-19 pandemic. Finally, we included treatment episodes occurring in various treatment settings, including ambulatory, nonintensive and intensive outpatient facilities, and residential treatment facilities. Analyses were performed from November 2023 to April 2024 using Stata, version 18.0 (StataCorp LLC). A 2-sided *P* < .05 was considered statistically significant.

## Results

### Characteristics of the Study Sample

Our sample consisted of 29 981 treatment episodes that met our inclusion criteria. As shown in Table 1, most treatment episodes were among non-Hispanic White individuals (74.4%), those aged 25 to 34 years (63.7%), and those who were never married (54.5%). Treatment episodes among other race and ethnicity categories included Asian, Pacific Islander, Native Hawaiian, or Other Pacific Islander (0.6%); Alaska Native or American Indian (1.8%); Hispanic (10.6%); non-Hispanic Black (6.6%); other single race or missing (3.6%); and 2 or more races (2.2%). Nearly half of all treatment episodes were among individuals who had completed high school (45.7%) and those who were either unemployed (43.5%) or not in the labor force (39.9%). Nearly three-quarters of episodes (71.8%) were among individuals living independently prior to admission, and about two-thirds (63.8%) were among those who had 1 or more prior treatment episodes. Most treatment episodes occurred in states that expanded Medicaid (84.2%) and those that had no child maltreatment law (70.5%).

**Table 1.** Admission Characteristics of Treatment Episodes Among Pregnant Individuals With a Primary OUD Diagnosis Admitted to Ambulatory, Nonintensive Outpatient Facilities

Characteristic	Treatment episodes			Unweighted SMD
	Total, No. (%) (N = 29 981)	With MOUD, No./total No. (%) (n = 19 884)	Without MOUD, No./total No. (%) (n = 9187)	
Age group, y				
15-24	6742 (22.5)	4149/6587 (63.0)	2438/6587 (37.0)	−0.136
25-34	19 106 (63.7)	12 851/18 512 (69.4)	5661/18 512 (30.6)	0.063
35-44	3771 (12.6)	2619/3622 (72.3)	1003/3622 (27.7)	0.068
≥45	362 (1.2)	265/350 (75.7)	85/350 (24.3)	0.037
Race and ethnicity				
Alaska Native, American Indian	544 (1.8)	421/542 (77.7)	121/542 (22.3)	0.060
Asian, Pacific Islander, Native Hawaiian, or Other Pacific Islander	176 (0.6)	124/174 (71.3)	50/174 (28.7)	0.010
Hispanic	3175 (10.6)	2313/3063 (75.1)	750/3063 (24.5)	0.113
Non-Hispanic Black	1994 (6.6)	1413/1962 (72.0)	549/1962 (28.0)	0.045
Non-Hispanic White	22 322 (74.4)	14 414/21 620 (66.7)	7206/21 620 (33.3)	−0.136
Other single race or missing^a^	1094 (3.6)	693/1043 (66.4)	350/1043 (33.6)	−0.017
≥2 Races	676 (2.2)	506/667 (75.9)	161/667 (24.1)	0.053
Employment status				
Unemployed	13 045 (43.5)	7984/12 542 (63.4)	4558/12 542 (36.3)	−0.191
Not in labor force	11 951 (39.9)	8853/11 722 (75.5)	2869/11 722 (24.5)	0.272
Part-time	2244 (7.5)	1394/2193 (63.6)	799/2193 (36.4)	−0.064
Full-time	2368 (7.9)	1464/2277 (64.3)	813/2277 (35.7)	−0.055
Missing or unknown	373 (1.2)	189/337 (68.4)	148/337 (43.9)	−0.060
Educational level				
No high school	1113 (3.7)	723/1086 (66.6)	363/1086 (33.4)	−0.017
Some high school (grades 9-11)	6762 (22.6)	4647/6568 (70.6)	1921/6568 (29.3)	0.059
High school or GED	13 709 (45.7)	9055/13 269 (68.2)	4214/13 269 (31.8)	−0.007
Some college or vocational school, or college or advanced degree	7635 (25.5)	5088/7404 (68.7)	2316/7404 (31.3)	0.009
Missing	762 (2.5)	371/744 (49.9)	373/744 (50.1)	−0.139
Marital status				
Never married	16 345 (54.5)	10 953/15 721 (69.7)	4768/15 721 (30.3)	0.064
Now married	3247 (10.8)	2181/3155 (69.1)	974/3155 (30.9)	0.012
Separated or divorced	2924 (9.7)	1835/2789 (65.8)	854/2789 (34.2)	−0.039
Missing	7465 (24.9)	4915/7406 (66.4)	2491/7406 (33.6)	−0.055
Co-occurring mental health conditions				
Yes	11 945 (39.8)	7969/11 786 (67.6)	3817/11 786 (32.4)	−0.030
No	14 627 (48.8)	10 121/13 965 (72.5)	3844/13 965 (27.5)	0.181
Missing	3409 (11.4)	1794/3320 (54.0)	1526/3320 (46.0)	−0239
Living arrangement				
Experiencing homelessness	2389 (8.0)	1685/2333 (72.2)	648/2333 (27.8)	0.052
Dependent living^b^	5510 (18.4)	3744/5328 (70.3)	1584/5328 (29.7)	0.040
Independent living^c^	21 522 (71.8)	14 179/20 902 (67.8)	6723/20 902 (32.2)	−0.042
Missing	560 (1.9)	276/508 (54.3)	232/508 (45.7)	−0.084
Referral source				
Individual (including self-referral)	16 077 (53.6)	12 299/15 559 (79.1)	3260/15 559 (21.0)	0.529
Alcohol or drug use care practitioner	2546 (8.5)	1919/2523 (76.1)	604/2523 (23.9)	0.110
Other health care practitioner, school, or employer	3458 (11.5)	2396/3386 (70.8)	990/3386 (29.2)	0.040
Court or criminal justice	3886 (13.0)	1489/3769 (39.5)	2280/3769 (60.5)	−0.516
Other community referral	3482 (11.6)	1502/3315 (45.3)	1813/3315 (54.7)	−0.380
Missing	532 (1.8)	279/519 (53.8)	240/519 (46.2)	−0.092
Route of opioid use				
Injection	15 066 (50.2)	10 284/14 642 (70.2)	4358/14 642 (29.8)	0.086
Oral, smoking, inhalation, other, or missing	14 915 (49.7)	9600/14 429 (66.5)	4829/14 429 (33.5)	−0.086
Secondary substance use				
None	9656 (32.2)	6984/9338 (74.8)	2354/9338 (25.2)	0.203
Alcohol	1169 (3.9)	572/1138 (50.2)	566/1138 (49.7)	−0.170
Stimulants^d^	8834 (29.5)	5786/8659 (66.8)	7778/8659 (49.3)	−0.048
Marijuana	3283 (11.0)	1953/3201 (61.0)	1248/3201 (39.0)	−0.120
Opioids	3775 (12.6)	2720/3674 (74.0)	954/3674 (26.0)	0.100
Sedative-hypnotics^e^	1382 (4.6)	821/1343 (61.1)	522/1343 (38.9)	−0.074
Hallucinogens	37 (0.1)	23/37 (62.2)	14/37 (37.8)	−0.010
Other^f^	643 (2.1)	331/610 (54.3)	279/610 (45.7)	−0.095
Missing	1202 (4.0)	694/1071 (64.8)	377/1071 (35.2)	−0.031
Prior treatment episode				
None	9747 (32.5)	6020/9368 (64.3)	3348/9368 (35.7)	−0.132
≥1	19 128 (63.8)	13 205/18 694 (70.6)	5489/18 694 (29.4)	0.139
Missing	1106 (3.7)	659/1009 (65.3)	350/1009 (34.7)	−0.026
Census division				
US territories	44 (0.1)	33/44 (75.0)	11/44 (25.0)	0.012
New England	3110 (10.4)	2372/3055 (77.6)	683/3055 (22.4)	0.147
Middle Atlantic	6797 (22.7)	5514/6665 (82.7)	1151/6665 (17.3)	−0.363
East North Central	4213 (14.0)	2748/4138 (66.4)	1390/4138 (33.6)	−0.038
West North Central	1566 (5.2)	1200/1552 (77.3)	352/1552 (22.7)	0.099
South Atlantic	3374 (11.2)	1122/2815 (39.9)	1693/2815 (60.1)	−0.405
East South Central	3274 (10.9)	1535/3241 (47.4)	1706/3241 (52.6)	−0.348
West South Central	355 (1.2)	134/347 (38.6)	213/347 (61.4)	−0.152
Mountain	2458 (8.2)	1334/2442 (54.6)	1108/2442 (45.4)	−0.195
Pacific	4790 (16.0)	3892/4772 (81.6)	880/4772 (18.4)	0.273
Year				
2015	4653 (15.5)	3006/4623 (65.0)	1617/4623 (35.0)	−0.069
2016	4844 (16.2)	3261/4818 (67.7)	1557/4818 (32.3)	−0.015
2017	5304 (17.7)	3583/5271 (68.0)	1688/5271 (32.0)	−0.009
2018	4874 (16.3)	3045/4628 (65.7)	1593/4628 (34.4)	−0.055
2019	4105 (13.7)	2710/3843 (70.5)	1133/3843 (29.5)	0.038
2020	3213 (10.7)	2168/3028 (71.6)	860/3028 (28.4)	0.050
2021	2988 (10.0)	2111/2850 (74.1)	739/2850 (25.9)	0.086
Treatment episode in Medicaid expansion state				
Yes	25 260 (84.2)	17 907/24 924 (71.9)	7017/24 924 (28.2)	0.376
No	4721 (15.7)	1977/4147 (47.7)	2170/4147 (52.3)	−0.376
Treatment episode in state with child maltreatment law				
Yes	8833 (29.5)	4143/8179 (50.7)	4036/8179 (49.4)	−0.507
No	21 148 (70.5)	15 741/20 892 (75.3)	5151/20 892 (24.7)	0.507

Abbreviations: GED, General Educational Development; MOUD, medication for opioid use disorder; OUD, opioid use disorder; SMD, standardized mean difference.

^a^ Treatment Episode Data Set-Discharges reports other single race as those not identified as American Indian or Alaska Native, Asian, Black or African American, Native Hawaiian or Other Pacific Islander, or White.

^b^ Supervised setting such as a residential institution, halfway house, or group home.

^c^ Living alone or with others in a private residence and capable of self-care.

^d^ Cocaine or crack, methamphetamine, or other amphetamines.

^e^ Benzodiazepines, other tranquilizers, barbiturates, or other sedatives or hypnotics.

^f^ Inhalants, over-the-counter medications, or substances marked as “other.”

**Figure 1.**
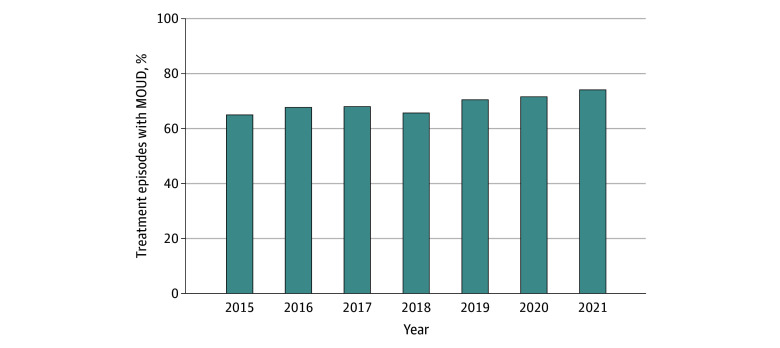
Changes in the Percentage of Treatment Episodes With Medications for Opioid Use Disorder (MOUD) Among Pregnant Individuals with a Primary OUD Diagnosis Admitted to Ambulatory, Nonintensive Outpatient Facilities From 2015 to 2021

Of 29 981 treatment episodes that met our inclusion criteria, a total of 29 071 were included in the analysis for MOUD, as 910 episodes had missing data for this variable. Across all study years, about two-thirds of all treatment episodes included MOUD (19 884 [68.4%]). MOUD inclusion in the treatment episode for pregnant individuals with OUD increased by 9.1 percentage points, from 65.0% in 2015 to 74.1% in 2021 (Figure 1).

There were statistically significant differences across all sociodemographic and treatment-related characteristics between treatment episodes with vs without MOUD. The proportion of treatment episodes with MOUD was higher among older individuals: 69.4% for individuals 25 to 34 years of age, 72.3% for individuals 35 to 44 years of age, and 75.7% for individuals 45 years or older compared with 63.0% for individuals 15 to 24 years of age (Table 1). The proportion was also higher among non-Hispanic Black (72.0%) and Hispanic (75.1%) individuals compared with non-Hispanic White individuals (66.7%). Compared with treatment episodes among persons referred by an individual or self-referred (79.1%), the proportion of episodes with MOUD was lowest for those referred from court or criminal justice settings (39.5%) and other community referrals (45.3%). The proportion of treatment episodes with MOUD was also greater for those with 1 or more prior episodes compared with no prior episodes (70.6% vs 64.3%). Middle Atlantic (82.7%), Pacific (81.6%), and New England (77.6%) census divisions had the highest proportions of treatment episodes with MOUD. A greater proportion of treatment episodes in states that expanded Medicaid included MOUD (71.9% vs 47.7%), while a lower proportion of episodes in states with child maltreatment laws included MOUD (50.7% vs 75.3%). The results of the logistic regression model of the characteristics associated with MOUD inclusion in the treatment plan are shown in eTable 1 in Supplement 1. Standardized mean differences across all covariates after propensity score weighting was less than 0.2, indicating good covariate balance (eTable 2 in Supplement 1).

### MOUD and Treatment Retention

In the IPT-weighted logistic regression model, treatment episodes with MOUD were associated with greater odds of treatment retention longer than 6 months compared with treatment episodes without MOUD (adjusted odds ratio [AOR], 1.86 [95% CI, 1.72-2.01]) (Table 2). MOUD was also positively associated with odds of 6-month treatment retention in all sensitivity analyses (eTable 3 in Supplement 1). The OR of an unmeasured confounder (E-value) would need to be 4.59 to explain away the association between MOUD and treatment retention. We found a significant interaction by referral source, with individuals receiving MOUD referred by other health care practitioners (AOR, 0.79 [95% CI, 0.64-0.98]) or the court and criminal justice system (AOR, 0.57 [95% CI, 0.47-0.68]) having lower odds of 6-month treatment retention compared with those referred by an individual or self-referred.

**Table 2.** Association Between MOUD Inclusion in Treatment Plan and Treatment Retention Longer Than 6 Months for Pregnant Individuals With a Primary OUD Diagnosis Admitted to Ambulatory, Nonintensive Outpatient Facilities^a^

Characteristic	OR (95% CI)	*P* value
MOUD in treatment episode	1.86 (1.72-2.01)	<.001
Age group, y		
25-34	1 [Reference]	NA
15-24	1.05 (0.97-1.16)	.22
35-44	0.96 (0.84-1.09)	.18
≥45	1.60 (1.08-2.38)	.04
Race and ethnicity		
Alaska Native or American Indian	0.94 (0.67-1.32)	.71
Asian, Pacific Islander, Native Hawaiian, or Other Pacific Islander	0.91 (0.55-1.52)	.72
Hispanic	0.89 (0.78-1.00)	.07
Non-Hispanic Black	0.85 (0.72-1.00)	.05
Non-Hispanic White	1 [Reference]	NA
Other single race or missing^b^	1.14 (0.91-1.41)	.25
≥2 Races	1.56 (1.14-2.12)	.005
Employment status		
Full-time	1 [Reference]	NA
Part-time	1.13 (0.95-1.35)	.16
Unemployed	0.87 (0.75-1.00)	.04
Not in labor force	0.98 (0.85-1.13)	.80
Missing or unknown	1.01 (0.69-1.48)	.95
Educational level		
High school or GED	1 [Reference]	NA
No high school	1.08 (0.88-1.32)	.45
Some high school (grades 9-11)	1.08 (0.98-1.20)	.12
Some college or vocational school, or college or advanced degree	1.15 (1.05-1.26)	.003
Missing	1.20 (0.97-1.48)	.09
Marital status		
Now married	1 [Reference]	NA
Separated or divorced	0.95 (0.79-1.13)	.54
Never married	0.82 (0.71-0.93)	.002
Missing	0.95 (0.79-1.14)	.60
Co-occurring mental health condition		
No	1 [Reference]	NA
Yes	0.89 (0.81-0.97)	.01
Missing	1.38 (1.18-1.61)	<.001
Living arrangement		
Independent living^c^	1 [Reference]	NA
Dependent living^d^	0.76 (0.68-0.84)	<.001
Experiencing homelessness	0.73 (0.62-0.86)	<.001
Missing	0.64 (0.46-0.90)	.01
Referral source		
Individual (including self-referral)	1 [Reference]	NA
Alcohol or drug use care practitioner	1.13 (0.98-1.29)	.10
Other health care practitioner, school, or employer	1.15 (1.02-1.30)	.02
Court or criminal justice	0.82 (0.74-0.91)	<.001
Other community referral	0.81 (0.73-0.91)	<.001
Missing	1.57 (1.23-2.00)	<.001
Route of opioid use		
Oral, smoking, inhalation, other, or missing	1 [Reference]	NA
Injection	0.95 (0.88-1.03)	.23
Secondary substance use		
None	1 [Reference]	NA
Alcohol	1.16 (0.97-1.38)	.10
Stimulant^e^	0.78 (0.71-0.86)	<.001
Marijuana	1.13 (1.02-1.26)	.06
Opioids	1.17 (1.02-1.34)	.03
Sedative-hypnotics^f^	0.94 (0.79-1.13)	.51
Hallucinogens	0.80 (0.32-1.98)	.62
Other^g^	0.81 (0.59-1.12)	.21
Missing	1.26 (1.02-1.54)	.03
Prior treatment episodes		
None	1 [Reference]	NA
≥1	1.10 (1.01-1.20)	.03
Treatment episode in Medicaid expansion state	0.74 (0.66-0.84)	<.001
Treatment episode in state with child maltreatment law	0.95 (0.85-1.07)	.44

Abbreviations: GED, General Educational Development; MOUD, medication for opioid use disorder; NA, not applicable; OR, odds ratio; OUD, opioid use disorder.

^a^ Inverse probability of treatment–weighted logistic regression models were used; the models were also adjusted for census division and year fixed effects.

^b^ Treatment Episode Data Set-Discharges reports other single race as individuals not identified as American Indian or Alaska Native, Asian, Black or African American, Native Hawaiian or Other Pacific Islander, or White.

^c^ Living alone or with others in a private residence and capable of self-care.

^d^ Supervised setting such as a residential institution, halfway house, or group home.

^e^ Cocaine or crack, methamphetamine, or other amphetamines.

^f^ Benzodiazepines, other tranquilizers, barbiturates, or other sedatives or hypnotics.

^g^ Inhalants, over-the-counter medications, or substances marked as “other.”

**Figure 2.**
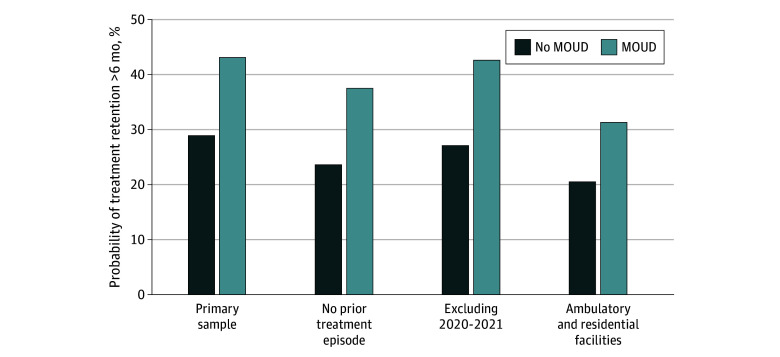
Estimated Probabilities of Treatment Retention Longer Than 6 Months Among Treatment Episodes With vs Without Medications for Opioid Use Disorder (MOUD) in the Primary Sample and in Sensitivity Analyses Estimated probabilities were calculated using inverse probability of treatment–weighted logistic regression models. The primary sample included treatment episodes in ambulatory, nonintensive facilities. Sensitivity analyses of individuals with no prior treatment episode and exclusion of COVID-19 pandemic years 2020 and 2021 were conducted in the primary sample.

Translating these results to estimated probabilities, we estimated a 14.2 percentage point greater probability of treatment retention for longer than 6 months among episodes with MOUD (43.1%) compared with those without it (28.9%) (Figure 2). When including all ambulatory and residential treatment settings, we estimated that MOUD inclusion in the treatment plan was associated with a 10.8 percentage point greater probability of treatment retention.

## Discussion

Using the most recently available national data on discharges from publicly funded substance use treatment facilities, we found that inclusion of evidence-based MOUD in treatment episodes among pregnant individuals with OUD increased by 9.1 percentage points from 2015 to 2021. A total of 68.4% of treatment episodes among pregnant individuals included the gold standard of care for OUD during a period when fentanyl and other HPSOs substantially increased the lethality of OUD.^28,29^ Treatment episodes involving MOUD were also significantly associated with greater odds of treatment retention for over 6 months. However, despite retention constituting a key element of the OUD cascade-of-care framework,^10^ retention rates remained low for this high-risk population.

This analysis contributes the most up-to-date evidence, to our knowledge, on treatment retention among pregnant individuals discharged from specialty substance use treatment facilities. Our findings are consistent with prior research in this population. For example, Nguemeni Tiako et al^17^ evaluated specialty substance use treatment program discharges from 2013 to 2017 and found a significant increase in treatment retention among treatment episodes that included MOUD (37.8% vs 16.6%). The MOUD retention rates observed in our study are also consistent with the 30% to 50% retention rates observed across most treatment settings for both pregnant and nonpregnant populations with OUD.^30,31,32,33,34,35^ Notably, retention is generally higher for individuals receiving methadone,^32,33,36^ although our dataset did not specify the type of MOUD provided to pregnant individuals. While the retention rate in our study was suboptimal given the lethality of OUD and the particular importance of treatment during pregnancy, it is comparable to rates observed in other chronic diseases, such as type 2 diabetes, for which retention is similarly challenging and influenced by psychosocial and socioeconomic barriers.^37,38,39^ Our findings add to the existing literature by using more recent data from years dominated by HPSOs. Furthermore, by applying IPT weights, our analysis helped to account for the differences between individuals receiving vs not receiving MOUD.^40^

Treating pregnant individuals with gold standard MOUD to improve OUD treatment retention is critical given the proliferation of HPSOs in the US illicit drug supply.^28,29^ Among pregnant individuals with OUD, there has been a large increase in pregnancy-associated overdose deaths involving HPSOs.^3^ Our findings add to the literature on the association of MOUD with treatment retention for pregnant individuals with OUD in an era of pervasive fentanyl availability. Despite the significant benefits of MOUD, our study found that only 68.4% of treatment episodes among pregnant individuals seen at treatment facilities nationwide had MOUD included in their treatment plan from 2015 to 2021. Pregnancy represents a critical window of opportunity when individuals are often motivated to engage in treatment.^41^ The benefits of MOUD span not only the prenatal period but also post partum. Notably, most pregnancy-associated overdose events occur during the first year post partum, and MOUD receipt during pregnancy is associated with reductions in overdose during the postpartum period.^3,42^ Given that shorter durations of MOUD use during pregnancy are associated with MOUD discontinuation post partum, improving treatment retention during pregnancy to reduce postpartum overdose rates is critical.^9,43^

Despite the significant benefits of MOUD among pregnant individuals, treatment facilities continue to provide these evidence-based treatments at low rates.^44,45,46^ Even among substance use treatment facilities with specialized programs for pregnant and postpartum individuals, which theoretically should provide the most comprehensive services for this population, less than 50% offer MOUD.^47^ Among facilities that offer MOUD, certain factors are associated with reduced MOUD receipt among pregnant populations. These include being younger, being referred from nonindividual settings (eg, carceral system), having no prior treatment episode, and receiving treatment outside ambulatory, nonintensive facilities—all of which are negatively associated with MOUD receipt.^12,16,17,48^ Future research should focus on identifying effective implementation strategies to promote widespread adoption of evidence-based MOUD among pregnant individuals receiving OUD treatment in specialty treatment settings. Our study also provided up-to-date findings on the association between state-level policies and MOUD receipt among pregnant individuals. Consistent with the results of an older study that examined 2012 TEDS data, we found that treatment episodes in states with child maltreatment laws had lower odds of MOUD inclusion in the treatment plan.^49^ Prior qualitative literature highlighted fears about the use of MOUD and scrutiny from the child welfare system resulting in potential custody loss.^50^ Our study provides further suggestive evidence that child maltreatment laws may inhibit MOUD uptake in this population.

### Limitations

Our study should be considered in the context of several limitations. First, the TEDS-D dataset does not capture treatment episodes for individuals who have not yet been discharged. As a result, we may have underestimated retention for those with long-term treatment episodes extending over multiple years, capturing only shorter-term episodes of care. However, since most individuals with OUD who initiate MOUD are not retained in treatment beyond 6 months,^51,52^ we believe our study captured the majority of treatment episodes. Additionally, TEDS data do not allow researchers to follow up individuals across treatment episodes or health care practitioners. For example, if an individual who switched practitioners or treatment facilities within 6 months of admission was still receiving care, they would be considered discharged from treatment in our data. This may have led to potential mismeasurement of treatment retention in our study. However, we conducted a sensitivity analysis excluding individuals with a prior treatment episode, which produced similar results as our primary analysis. Second, some pregnant individuals may have received buprenorphine through office-based prescribers not captured in TEDS but were receiving other services through treatment facilities captured in TEDS. Therefore, we may have underestimated the proportion of individuals receiving MOUD. Third, the MOUD variable in TEDS does not distinguish between types of MOUD. While methadone has shown better retention rates compared with buprenorphine, we were unable to examine retention differences by MOUD type in our study.^36,53^ Furthermore, prior literature has shown that higher MOUD doses are associated with better treatment retention, particularly in the HPSO era, but our study lacked dosing data.^54,55^ Fourth, TEDS data do not provide information on the gestational age or trimester of pregnancy. Therefore, we were unable to determine whether we were measuring retention during pregnancy or the postpartum period. The postpartum period is a time of increased MOUD discontinuation,^56^ and this may have influenced treatment retention patterns in our study. Fifth, IPT-weighted models can only adjust for observed characteristics. There may be unobserved characteristics associated with both MOUD receipt and treatment retention that we did not capture in the IPT weights. However, our calculated E-value of 4.59 suggests that an unmeasured confounder would need to have a strong correlation with both MOUD and retention to explain away the observed treatment effect.

## Conclusions

In this cross-sectional study of treatment episodes among pregnant individuals in publicly funded, ambulatory, nonintensive outpatient settings, we found that MOUD inclusion was associated with significant improvements in treatment retention during the HPSO era. Yet, many pregnant individuals still did not receive MOUD as part of their care. Even among those who did, the majority did not remain in treatment for the recommended minimum of 6 months. Our findings underscore the importance of policy and practice interventions to increase MOUD receipt and retention to improve OUD-related outcomes in this vulnerable population.

## Notes

Supplement 1. **eAppendix 1.** Description of Covariates **eAppendix 2.** Details on Inverse Probability of Treatment Weighting Procedure **eTable 1.** Characteristics Associated With MOUD Inclusion in Treatment Plan for Pregnant Individuals With Primary OUD Diagnosis Admitted to Ambulatory, Non-Intensive Outpatient Facilities **eTable 2.** Standardized Mean Differences (SMD) Before and After Inverse Probability of Treatment Weighting for the Analysis of MOUD and Treatment Retention **eTable 3.** Results of Sensitivity Analyses Examining the Association Between MOUD Inclusion in Treatment Plan and Treatment Retention >6 Months for Pregnant Individuals With Primary OUD Diagnosis Admitted to Ambulatory, Non-Intensive Outpatient Facilities Using Inverse Probability of Treatment Weighting

Supplement 2.
Data Sharing Statement
