# Supplementary material for: Medication for Opioid Use Disorder and Treatment Retention Among Pregnant Individuals
Source: JAMA Netw Open. 2025 Apr 21;8(4):e256069. doi: 10.1001/jamanetworkopen.2025.6069 (PMC12013350; doi:10.1001/jamanetworkopen.2025.6069)
Supplement: Supplement 1. — eAppendix 1. Description of Covariates eAppendix 2. Details on Inverse Probability of Treatment Weighting Procedure eTable 1. Characteristics Associated With MOUD Inclusion in Treatment Plan for Pregnant Individuals With Primary OUD Diagnosis Admitted to Ambulatory, Non-Intensive Outpatient Facilities eTable 2. Standardized Mean Differences (SMD) Before and After Inverse Probability of Treatment Weighting for the Analysis of MOUD and Treatment Retention eTable 3. Results of Sensitivity Analyses Examining the Association Between MOUD Inclusion in Treatment Plan and Treatment Retention >6 Months for Pregnant Individuals With Primary OUD Diagnosis Admitted to Ambulatory, Non-Intensive Outpatient Facilities Using Inverse Probability of Treatment Weighting [file jamanetwopen-e256069-s001.pdf]

Supplementary Online Content

Ganetsky VS, Krawczyk N, Kennedy-Hendricks A. Medication for opioid use disorder and treatment retention among pregnant individuals. *JAMA Netw Open*. 2025;8(4):e256069. doi:10.1001/jamanetworkopen.2025.6069

eAppendix 1. Description of Covariates

eAppendix 2. Details on Inverse Probability of Treatment Weighting Procedure

eTable 1. Characteristics Associated With MOUD Inclusion in Treatment Plan for Pregnant Individuals With Primary OUD Diagnosis Admitted to Ambulatory, Non-Intensive Outpatient Facilities

eTable 2. Standardized Mean Differences (SMD) Before and After Inverse Probability of Treatment Weighting for the Analysis of MOUD and Treatment Retention

eTable 3. Results of Sensitivity Analyses Examining the Association Between MOUD Inclusion in Treatment Plan and Treatment Retention >6 Months for Pregnant Individuals With Primary OUD Diagnosis Admitted to Ambulatory, Non-Intensive Outpatient Facilities Using Inverse Probability of Treatment Weighting

This supplementary material has been provided by the authors to give readers additional information about their work.

© 2025 Ganetsky VS et al. *JAMA Network Open*.

eAppendix 1. Description of Covariates

Covariates hypothesized as potential confounders of the relationship between MOUD inclusion in the treatment episode and retention included the following: sociodemographic characteristics including age (15-24, 25-34, 35-44, ≥45), race/ethnicity (White, non-Hispanic; Black, non-Hispanic; Alaska Native or American Indian; Asian, Pacific Islander, Native Hawaiian, or Other Pacific Islander; two or more races; other single race or missing), marital status (never married, now married, separated or divorces, missing), education level (no high school; some high school; high school/GED; some college, vocational school, or college/advanced degree; missing), employment status (unemployed, not in labor force, part-time, full-time, missing or unknown), and housing (experiencing homelessness, dependent living, independent living, missing); substance use, mental health, and treatment history including route of opioid administration (injection vs. oral, smoking, inhalation, other, or missing), secondary substance use (none, alcohol, stimulants, marijuana, opioids, sedative-hypnotics, hallucinogens, other, missing), co-occurring mental health conditions (yes, no, missing), and any prior treatment episode (yes/no); and treatment admission-related variables including referral source (individual; alcohol or drug use care provider; other healthcare provider, school, or employer; court or criminal justice; other community referral; missing) and treatment services provided at admission excluding detoxification (ambulatory, non-intensive outpatient; ambulatory intensive outpatient; rehabilitation/residential hospital or residential short-term; rehabilitation/residential long-term).

eAppendix 2. Details on Inverse Probability of Treatment Weighting Procedure

All episodes that did include MOUD were assigned a weight of one. To calculate IPT weights for treatment episodes not including MOUD, we first estimated logistic regression models with MOUD inclusion in the treatment plan as a function of sociodemographic, substance use, mental health, treatment history, treatment admission-related factors, state policy environment, Census region fixed effects, and year fixed effects (eTable 1). We then used the model estimated probabilities of MOUD inclusion in each treatment episode to construct individual weights by calculating the inverse of this probability. Under this approach, observations with higher estimated probabilities of MOUD inclusion in the treatment plan (i.e., with probabilities closer to one) had larger weights.

eTable 1. Characteristics Associated With MOUD Inclusion in Treatment Plan for Pregnant Individuals With Primary OUD Diagnosis Admitted to Ambulatory, Non-Intensive Outpatient Facilities[a]

Characteristic	MOUD in Treatment Episode OR (95% CI)	P value
Age (years)		
25-34	Reference	
15-24	0.74 (0.69-0.79)	<0.01
35-44	1.17 (1.07-1.29)	<0.01
≥45	1.05 (0.79-1.38)	0.75
Race/ethnicity		
White, non-Hispanic	Reference	
Black, non-Hispanic	1.29 (1.14-1.46)	<0.01
Hispanic	1.23 (1.11-1.36)	<0.01
Asian, Pacific Islander, Native Hawaiian, or Other Pacific Islander	1.02 (0.71-1.47)	0.90
Two or more races	1.50 (1.22-1.84)	<0.01
Other single race[b] or missing	1.16 (1.00-1.35)	0.05
Employment		
Full-time	Reference	
Part-time	0.95 (0.82-1.09)	0.46
Unemployed	1.08 (0.97-1.20)	0.17
Not in labor force	1.36 (1.21-1.52)	<0.01
Missing or unknown	0.90 (0.68-1.20)	0.48
Education		
High school/GED	Reference	
No high school	0.80 (0.69-0.93)	<0.01
Some high school (grades 9-11)	1.21 (1.12-1.30)	<0.01
Some college/vocational school, or college/advanced degree	0.97 (0.91-1.05)	0.48
Missing	0.61 (0.51-0.73)	<0.01
Marital status		
Now married	Reference	
Separated or divorced	1.00 (0.88-1.14)	0.66
Never married	1.04 (0.94-1.15)	0.42
Missing	0.48 (0.43-0.55)	<0.01
Co-occurring mental health condition		
No	Reference	
Yes	0.75 (0.70-0.80)	<0.01
Missing	0.82 (0.73-0.92)	<0.01
Living arrangement		
Independent living[c]	Reference	
Dependent living[d]	1.36 (1.25-1.47)	<0.01
Experiencing homelessness	1.19 (1.07-1.33)	<0.01
Missing	0.94 (0.76-1.16)	0.58
Referral source		
Individual (including self-referral)	Reference	
Alcohol/drug use care provider	0.69 (0.62-0.77)	<0.01
Other healthcare provider, school, or employer	0.80 (0.73-0.88)	<0.01
Court/criminal justice	0.16 (0.14-0.17)	<0.01
Other community referral	0.25 (0.23-0.28)	<0.01
Missing	0.47 (0.38-0.57)	<0.01

© 2025 Ganetsky VS et al. *JAMA Network Open*.

Route of opioid use		
Oral, smoking, inhalation, other, or missing	Reference	
Injection	1.16 (1.09-1.23)	<0.01
Secondary substance use		
None	Reference	
Alcohol	0.35 (0.30-0.40)	<0.01
Stimulants[e]	0.61 (0.57-0.66)	<0.01
Marijuana	0.55 (0.50-0.61)	<0.01
Opioids	1.11 (1.01-1.23)	0.04
Sedative-hypnotics[f]	0.59 (0.52-0.68)	<0.01
Hallucinogens	0.56 (0.25-1.23)	0.15
Other[g]	0.56 (0.46-0.67)	<0.01
Missing	1.11 (0.95-1.30)	0.20
Prior treatment episodes		
No prior episodes	Reference	
One or more prior episodes	1.17 (1.09-1.25)	<0.01
Treatment episode in Medicaid expansion state	1.61 (1.47-1.78)	<0.01
Treatment episode in state with child maltreatment law,	0.70 (0.64-0.77)	<0.01

Abbreviations: CI, confidence interval; GED, General Educational Development; MOUD, medication for opioid use disorder; OR, odds ratio; OUD, opioid use disorder

[a] Model was also adjusted for census division and year fixed effects

[b] TEDS reports on race using the Office of Management and Budget's (OMB) five minimum categories. Other single race includes those not identified as White, Black or African American, American Indian or Alaska Native, Asian, and Native Hawaiian or Other Pacific Islander. It also includes records from states that did not separate the race categories "Asian" and "Native Hawaiian or Other Pacific Islander."

[c] Living alone or with others in a private residence and capable of self-care

[d] Supervised setting such as a residential institution, halfway house, or group home

[e] Cocaine/crack, methamphetamine, or other amphetamines

[f] Benzodiazepines, other tranquilizers, barbiturates, or other sedatives/hypnotics

[g] Inhalants, over-the-counter medications, or those marked as "other"

© 2025 Ganetsky VS et al. *JAMA Network Open*.

eTable 2. Standardized Mean Differences (SMD) Before and After Inverse Probability of Treatment Weighting for the Analysis of MOUD and Treatment Retention

Characteristic	Unweighted SMD	Weighted SMD
Age in years		
15-24	-0.136	-0.036
25-34	0.062	0.007
35-44	0.068	0.032
≥45	0.037	0.008
Race/ethnicity		
White, non-Hispanic	-0.136	-0.051
Hispanic	0.113	0.046
Black, non-Hispanic	0.045	0.045
Other single race[a] or missing	-0.017	-0.023
Alaska Native, American Indian	0.060	0.006
Two or more races	0.053	0.009
Asian, Pacific Islander, Native Hawaiian, or Other Pacific Islander	0.010	-0.009
Employment		
Unemployed	-0.191	-0.046
Not in labor force	0.271	0.084
Part-time	-0.064	-0.019
Full-time	-0.055	-0.034
Missing or unknown	-0.060	-0.038
Education		
No high school	-0.017	-0.031
Some high school (grades 9-11)	0.059	0.036
High school/GED	-0.007	0.007
Some college/vocational school, or college/advanced degree	0.009	-0.009
Missing	-0.139	-0.055
Marital status		
Never married	0.064	-0.014
Now married	0.012	-0.023
Separated or divorced	-0.039	-0.025
Missing	-0.055	0.049
Co-occurring mental health conditions		
Yes	-0.030	-0.042
No	0.181	0.076
Missing	-0.239	-0.055
Living arrangement		
Experiencing homelessness	0.052	-0.012
Dependent living[b]	0.041	0.006
Independent living[c]	-0.042	0.020
Missing	-0.083	-0.057
Referral source		
Individual (including self-referral)	0.529	0.235
Alcohol/drug use care provider	0.110	0.018
Other healthcare provider, school, or employer	0.040	-0.008
Court/criminal justice	-0.516	-0.158
Other community referral	-0.380	-0.117
Missing	-0.092	-0.030
Route of opioid use		
Injection	0.086	0.035

© 2025 Ganetsky VS et al. *JAMA Network Open*.

Secondary substance use		
None	0.203	0.072
Alcohol	-0.170	-0.053
Stimulants[d]	-0.048	-0.004
Marijuana	-0.120	-0.046
Opioids	0.099	0.001
Sedative-hypnotics[e]	-0.074	-0.023
Hallucinogens	-0.010	-0.004
Other[f]	-0.095	-0.046
Missing	-0.031	0.003
Prior treatment episodes		
No prior episodes	-0.132	-0.027
One or more prior episodes	0.139	0.033
Missing	-0.026	-0.015
Census division		
US territories	0.012	-0.001
New England	0.147	-0.006
Middle Atlantic	0.363	0.132
East North Central	-0.038	-0.009
West North Central	0.099	0.039
South Atlantic	-0.404	-0.113
East South Central	-0.348	-0.114
West South Central	-0.152	-0.049
Mountain	-0.195	-0.076
Pacific	0.273	0.105
Year		
2015	-0.069	-0.017
2016	-0.015	-0.009
2017	-0.009	-0.008
2018	-0.055	-0.006
2019	0.038	-0.001
2020	0.050	0.005
2021	0.086	0.045
Treatment episode in Medicaid expansion state	0.376	0.122
Treatment episode in state with child maltreatment law	-0.507	-0.178

Abbreviations: GED, General Educational Development; MOUD, medication for opioid use disorder

[a] TEDS reports on race using the Office of Management and Budget's (OMB) five minimum categories. Other single race includes those not identified as White, Black or African American, American Indian or Alaska Native, Asian, and Native Hawaiian or Other Pacific Islander. It also includes records from states that did not separate the race categories "Asian" and "Native Hawaiian or Other Pacific Islander."

[b] Living alone or with others in a private residence and capable of self-care

[c] Supervised setting such as a residential institution, halfway house, or group home

[d] Cocaine/crack, methamphetamine, or other amphetamines

[e] Benzodiazepines, other tranquilizers, barbiturates, or other sedatives/hypnotics

[f] Inhalants, over-the-counter medications, or those marked as "other"

eTable 3. Results of Sensitivity Analyses Examining the Association Between MOUD Inclusion in Treatment Plan and Treatment Retention >6 Months for Pregnant Individuals With Primary OUD Diagnosis Admitted to Ambulatory, Non-Intensive Outpatient Facilities Using Inverse Probability of Treatment Weighting[a]

Characteristic	No Prior Treatment Episodes OR (95% CI)	Excluding Years 2020-2021 OR (95% CI)	Ambulatory and Residential Facilities OR (95% CI)
MOUD in treatment episode	1.94 (1.69-2.23)	2.00 (1.84-2.18)	1.77 (1.66-1.89)
Age (years)			
25-34	Reference	Reference	Reference
15-24	1.02 (0.88-1.19)	1.04 (0.95-1.14)	1.09 (1.01-1.18)
35-44	0.93 (0.74-1.17)	0.97 (0.84-1.13)	0.92 (0.82-1.03)
>=45	1.64 (0.80-3.35)	1.57 (1.08-2.28)	1.39 (1.03-1.87)
Race/ethnicity			
White, non-Hispanic	Reference	Reference	Reference
Black, non-Hispanic	0.84 (0.66-1.08)	0.81 (0.68-0.97)	0.84 (0.74-0.96)
Hispanic	0.87 (0.71-1.08)	0.89 (0.77-1.03)	0.89 (0.80-1.00)
Asian, Pacific Islander, Native Hawaiian, or Other Pacific Islander	0.76 (0.35-1.64)	0.75 (0.45-1.24)	0.87 (0.59-1.28)
Alaska Native, American Indian	0.69 (0.40-1.19)	0.95 (0.66-1.36)	1.34 (1.08-1.65)
Two or more races	1.51 (0.89-2.57)	1.53 (1.09-2.16)	1.03 (0.86-1.22)
Other single race[b] or missing	1.04 (0.68-1.59)	0.98 (0.77-1.25)	0.84 (0.74-0.96)
Employment			
Full-time	Reference	Reference	Reference
Part-time	0.99 (0.73-1.34)	1.11 (0.92-1.35)	1.12 (0.95-1.31)
Unemployed	0.75 (0.59-0.96)	0.89 (0.76-1.04)	0.86 (0.76-0.97)
Not in labor force	0.91 (0.70-1.17)	0.97 (0.83-1.15)	1.01 (0.89-1.15)
Missing or unknown	1.13 (0.54-2.36)	0.69 (0.47-1.02)	0.84 (0.63-1.12)
Education			
High school/GED	Reference	Reference	Reference
No high school	0.96 (0.65-1.42)	1.03 (0.85-1.26)	1.16 (0.98-1.38)
Some high school (grades 9-11)	1.00 (0.85-1.19)	1.12 (1.01-1.25)	1.09 (1.00-1.18)
Some college/vocational school, or college/advanced degree	1.12 (0.95-1.32)	1.16 (1.05-1.29)	1.17 (1.08-1.27)
Missing	1.05 (0.62-1.77)	1.28 (1.04-1.58)	1.22 (1.02-1.47)
Marital status			
Now married	Reference	Reference	Reference
Separated or divorced	1.13 (0.81-1.59)	0.99 (0.81-1.21)	1.02 (0.88-1.19)
Never married	0.82 (0.65-1.04)	0.79 (0.69-0.91)	0.86 (0.77-0.96)
Missing	0.90 (0.68-1.20)	1.02 (0.84-1.23)	1.07 (0.93-1.23)
Co-occurring mental health condition			
No	Reference	Reference	Reference
Yes	0.82 (0.69-0.97)	0.91 (0.82-1.01)	0.91 (0.85-0.99)
Missing	1.45 (1.10-1.91)	1.42 (1.20-1.68)	1.21 (1.08-1.36)
Living arrangement			
Independent living[c]	Reference	Reference	Reference
Dependent living[d]	0.78 (0.64-0.94)	0.72 (0.64-0.81)	1.28 (1.15-1.43)
Experiencing homelessness	0.67 (0.51-0.89)	0.72 (0.61-0.85)	1.04 (0.92-1.18)
Missing	0.76 (0.45-1.27)	0.54 (0.40-0.72)	0.95 (0.75-1.20)
Referral source			
Individual (including self-referral)	Reference	Reference	Reference

© 2025 Ganetsky VS et al. *JAMA Network Open*.

Alcohol/drug use care provider	1.23 (0.92-1.64)	1.10 (0.94-1.27)	1.11 (0.99-1.25)
Other healthcare provider, school, or employer	1.11 (0.90-1.36)	1.11 (0.97-1.26)	1.18 (1.06-1.31)
Court/criminal justice	0.74 (0.61-0.91)	0.83 (0.74-0.93)	0.94 (0.87-1.03)
Other community referral	0.78 (0.65-0.95)	0.84 (0.74-0.95)	0.84 (0.76-0.92)
Missing	2.01 (1.33-3.02)	1.45 (1.11-1.89)	1.52 (1.26-1.84)
Route of opioid use			
Oral, smoking, inhalation, other, or missing	Reference	Reference	Reference
Injection	0.85 (0.74-0.98)	0.92 (0.85-1.01)	0.93 (0.87-0.99)
Secondary substance use			
None	Reference	Reference	Reference
Alcohol	0.83 (0.59-1.17)	1.12 (0.93-1.34)	1.14 (0.99-1.32)
Stimulants[e]	0.73 (0.62-0.87)	0.77 (0.69-0.86)	0.82 (0.75-0.89)
Marijuana	1.03 (0.84-1.27)	1.11 (0.97-1.26)	1.14 (1.02-1.26)
Opioids	1.07 (0.85-1.34)	1.16 (1.01-1.35)	1.16 (1.03-1.31)
Sedative-hypnotics[f]	0.79 (0.56-1.13)	0.86 (0.71-1.04)	0.92 (0.79-1.07)
Hallucinogens	4.81 (0.09-266.31)	0.70 (0.26-1.88)	0.91 (0.37-2.23)
Other[g]	0.37 (0.23-0.60)	0.85 (0.60-1.21)	0.73 (0.56-0.93)
Missing	1.03 (0.74-1.45)	1.24 (1.00-1.55)	1.28 (1.09-1.52)
Prior treatment episodes			
No prior episodes	-	Reference	Reference
One or more prior episodes	-	1.13 (1.02-1.24)	1.09 (1.02-1.18)
Admission service			
Ambulatory, non-intensive outpatient	-	-	Reference
Rehabilitation/residential hospital or residential short-term	-	-	0.07 (0.06-0.08)
Rehabilitation/residential long-term	-	-	0.39 (0.35-0.43)
Ambulatory intensive outpatient	-	-	0.45 (0.42-0.50)
Treatment episode in Medicaid expansion state	0.73 (0.58-0.93)	0.78 (0.68-0.89)	0.76 (0.69-0.83)
Treatment episode in state with child maltreatment law	1.22 (0.98-1.52)	1.09 (0.95-1.25)	0.98 (0.89-1.08)

Abbreviations: CI, confidence interval; GED, General Educational Development; MOUD, medication for opioid use disorder; OR, odds ratio; OUD, opioid use disorder

[a] Model was also adjusted for census division and year fixed effects

[b] TEDS reports on race using the Office of Management and Budget's (OMB) five minimum categories. Other single race includes those not identified as White, Black or African American, American Indian or Alaska Native, Asian, and Native Hawaiian or Other Pacific Islander. It also includes records from states that did not separate the race categories "Asian" and "Native Hawaiian or Other Pacific Islander."

[c] Living alone or with others in a private residence and capable of self-care

[d] Supervised setting such as a residential institution, halfway house, or group home

[e] Cocaine/crack, methamphetamine, or other amphetamines

[f] Benzodiazepines, other tranquilizers, barbiturates, or other sedatives/hypnotics

[g] Inhalants, over-the-counter medications, or those marked as "other"

© 2025 Ganetsky VS et al. *JAMA Network Open.*
