# Supplementary material for: Medication for Opioid Use Disorder and Treatment Retention Among Pregnant Individuals
Source: JAMA Netw Open. 2025 Apr 21;8(4):e256069. doi: 10.1001/jamanetworkopen.2025.6069 (PMC12013350; doi:10.1001/jamanetworkopen.2025.6069)
Supplement: Supplement 2. — Data Sharing Statement [file jamanetwopen-e256069-s002.pdf]

# Data Sharing Statement

Ganetsky. Medication for Opioid Use Disorder and Treatment Retention Among Pregnant Individuals. *JAMA Netw Open*. Published April 21, 2025.
doi:10.1001/jamanetworkopen.2025.6069

**Data**
**Data available:** No

**Additional Information**
**Explanation for why data not available:** Data will be made available upon request.
